# Silencing of type Iγ phosphatidylinositol phosphate kinase suppresses ovarian cancer cell proliferation, migration and invasion

**DOI:** 10.3892/or.2021.7982

**Published:** 2021-02-18

**Authors:** Siyu Cao, Chunhua Chen, Junli Xue, Yan Huang, Xiaofeng Yang, Kun Ling

Oncol Rep 38: 253-262, 2017; DOI: 10.3892/or.2017.5670

Subsequently to the publication of the above paper, the authors have drawn to our attention that the middle panel in Fig. 3B, representing the migration of PIPKIγ-depleted cells (PIPKIγ-1), was inadvertently mixed up with the left panel of control cells (siRNA Ctrl). The results presented in Fig. 3D, however, were quantified based on the original images from three independent experiments, each containing five randomly picked microscopic fields.

The authors were able to re-examine the original data files and retrieve the correct data panels. The revised version of Fig. 3, featuring the correct data for the ‘PIPKIγ-1’ panel in Fig. 3B, is shown below. Note that the error made inadvertently with the selection of the representative image for PIPKIγ-1 in Fig. 3B did not affect the overall conclusions reported for this experiment. The authors are grateful to the Editor of *Oncology Reports* for allowing them the opportunity to publish this Corrigendum, and apologize to the readership for any inconvenience caused.

## Figures and Tables

**Figure 3.**
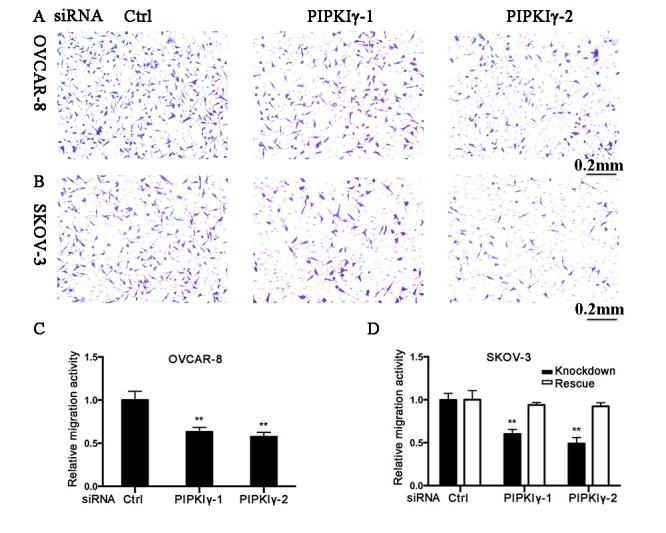
Loss of PIPKIγ suppresses the migration of epithelial ovarian cancer cells. Migration assay was performed using modified Boyden chambers in triplicates using OVCAR-8 (A) or SKOV-3 (B) cells transfected with the indicated siRNAs (control, PIPKIγ-1 and PIPKIγ-2). (A and B) Cells migrating across the membrane were fixed and stained, then imaged under a microscope. (C and D) Cells imaged in A and B were counted in five random fields under ×20 magnification and averaged, and then statistically analyzed from three independent experiments and plotted. (D) Rescue experiments were conducted using SKOV-3 cells by introducing the expression of siRNA-resistant PIPKIγ isoform 1 and 2 by transient transfection, followed by transfection of control or PIPKIγ-specific siRNAs. Then cells were subjected to migration assay and quantified as described above. Data are presented mean ± SD. **P<0.01. PIPKIγ, type Iγ phosphatidylinositol phosphate kinase.

